# 2022 *BMC Ecology and Evolution* image competition: the winning images

**DOI:** 10.1186/s12862-022-02049-y

**Published:** 2022-08-19

**Authors:** Jennifer Harman, Christy A. Hipsley, Luke M. Jacobus, David A. Liberles, Josef Settele, Arne Traulsen

**Affiliations:** 1 BMC, London, UK; 2 University of Copenhagen, Copenhagen, Denmark; 3 Indiana University-Purdue University Columbus (IUPUC), Columbus, IN USA; 4 Temple University, Pennsylvania, USA; 5 Helmholtz-Centre for Environmental Research-UFZ, Leipzig, Germany; 6 Max-Planck Institute for Evolutionary Biology, Schleswig-Holstein, Germany

## Abstract

In 2022, researchers from around the world entered the *BMC Ecology and Evolution* photography competition. The contest produced a spectacular collection of photographs that capture the wonder of the natural world and the growing need to protect it as the human impact on the planet intensifies. This editorial celebrates the winning images selected by the Editor of *BMC Ecology and Evolution* and senior members of the journal’s editorial board.

**Supplementary Information:**

The online version contains supplementary material available at 10.1186/s12862-022-02049-y.

## Introduction

We are delighted to announce the winning images of the *BMC Ecology and Evolution* photography competition. Like in previous years [1–8], the researchers who participated in this annual contest have, once again, produced a spectacular collection of images. The competition attracted entries from ecologists and evolutionary biologists from around the world eager to use their creativity to highlight the wonder of nature, the challenges facing our planet and their research. *BMC Ecology and Evolution* invited anyone affiliated with a research institution to submit to one of the following four categories: ‘Relationships in Nature’, ‘Biodiversity under Threat’, ‘Life Close Up’ and ‘Research in Action’.

Our Senior Editorial Board Members lent their expertise to judge the submissions, selecting the overall winner, best image, and runner-up from each category. The board members considered the scientific story behind the photos in addition to their artistic judgement.

## Overall winner

**Fig. 1:**
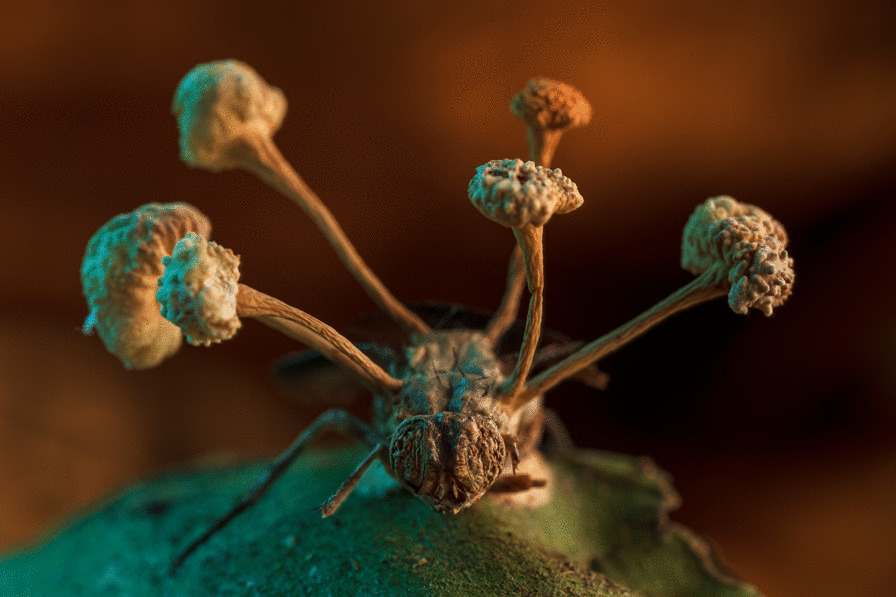
The story of a conquest. The fruiting body of a parasitic fungus erupts from the body of its victim. Attribution: Roberto García-Roa

The overall winner captures something like out of science fiction—the fruiting body of a parasitic fungus erupting from the body of a fly. Roberto García-Roa, an evolutionary biologist and conservation photographer affiliated with the University of Valencia (Spain) and Lund University (Sweden), captured this unsettling image in the Peruvian jungle of Tambopata. Roberto explains that “spores of the so-called ‘Zombie’ fungus (e.g. genera *Ophiocordyceps*) infect arthropods by infiltrating their exoskeleton and minds. As a result, parasitized hosts are compelled to migrate to a more favourable location for the fungus’s growth. Here, they await death, at which point the fungus feeds on its host to produce fruiting bodies full of spores that will be jettisoned to infect more victims—a conquest shaped by thousands of years of evolution.” Senior Editorial Board Member Christy Anna Hipsley comments that this image depicting a parasite-host interaction “has a depth and composition that conveys life and death simultaneously—an affair that transcends time, space, and even species. The death of the fly gives life to the fungus” (Additional file 1; Fig. S1) (Fig. 1).

## Relationships in nature: best in category

**Fig. 2:**
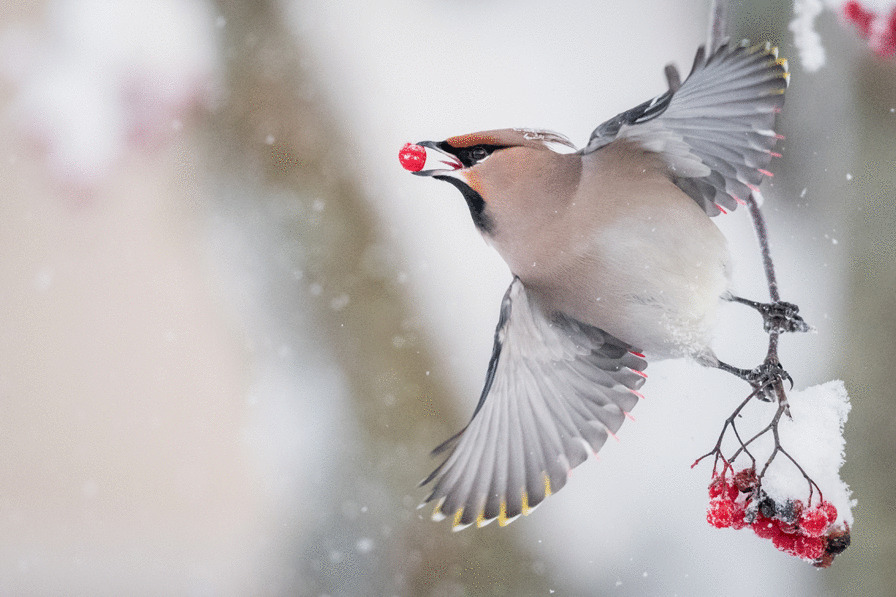
Gone with the berry. Flying under the influence—a waxwing feasts on fermented rowan berries. Attribution: Alwin Hardenbol

**Fig. 3:**
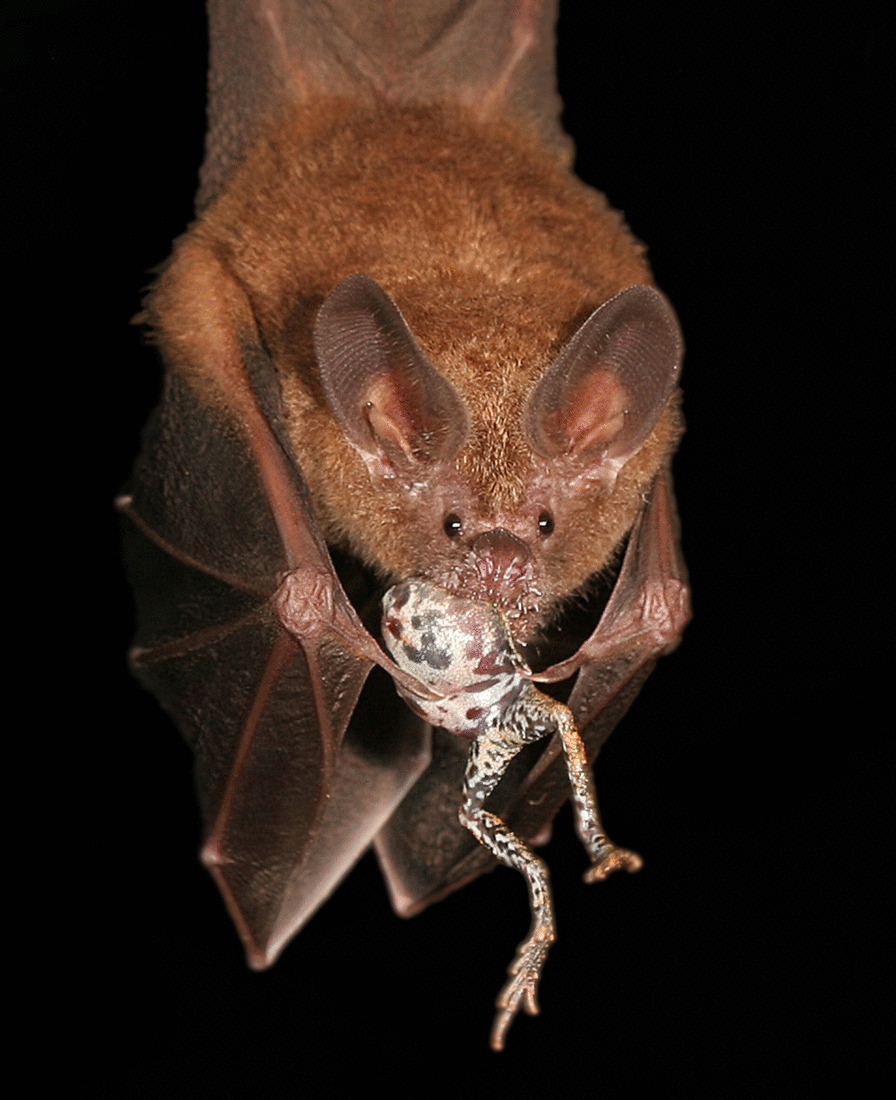
Trachops & Tungara. A bat locates its dinner via tuning into a frog’s broadcast to attract a mate. Attribution: Alexander T. Baugh

The winning image for this category, which beautifully captures a plant-frugivore relationship, was submitted by Alwin Hardenbol. Alwin is a Postdoctoral Researcher at the University of Eastern Finland with a background in forest ecology and a talent for nature photography. Alwin said: "Bohemian Waxwings (*Bombycilla garrulus*) have a strong relationship with rowan trees due to the berries they produce. This plant-frugivore interaction is so strong that this bird species will migrate based on the presence of rowan berries. In years when there are many rowan berries in Finland, where I took this picture, the Waxwings may barely migrate. However, in other years, they can reach Western, Eastern, and Central Europe in huge numbers until they reach their beloved rowan berries. Waxwings can eat several hundred berries per day exceeding double their own weight, largely in part due to the low nutritional values of berries, with the exception of sugars. While this relationship is highly beneficial for seed dispersal, it does not come without a cost for the birds. As the berries become overripe, they start to ferment and produce ethanol which gets Waxwings intoxicated, sometimes leading to trouble for the birds, even death. Unsurprisingly, Waxwings have evolved to have a relatively large liver to deal with their inadvertent alcoholism." Senior Editorial Board Member Luke Jacobus comments that "this easily recognizable image evokes an immediate response from the viewer, clearly communicating action, reaction and interaction, including biotic and abiotic players. The contrasting colours and carefully crafted composition capture a fleeting but personal instant in which it seems the waxwing responds to the viewer too. This image captures an ecological relationship forged through creative evolutionary forces” (Additional file 2; Fig. S2) (Figs. 2, 3).

## Relationships in nature: runner-up

Alexander T. Baugh, a behavioural biologist at Swarthmore College, USA, captured the runner-up depicting a predator–prey relationship at the Smithsonian Tropical Research Institute in Panama. Alexander comments that “This image illustrates how natural and sexual selection can be at odds. A male tungara frog (*Physalalamus pustulosus*) makes a tasty meal for a hungry fringe-lipped bat (*Trachops cirrhosis*) that detected and localised the frog by listening to the mating call.” These bats are specialised to hunt frogs, their hearing is adapted to their low frequency mating calls and their salivary glands may neutralise the toxins in the skin of poisonous prey (Additional file 3; Fig. S3).

## Biodiversity under threat: best in category

**Fig. 4:**
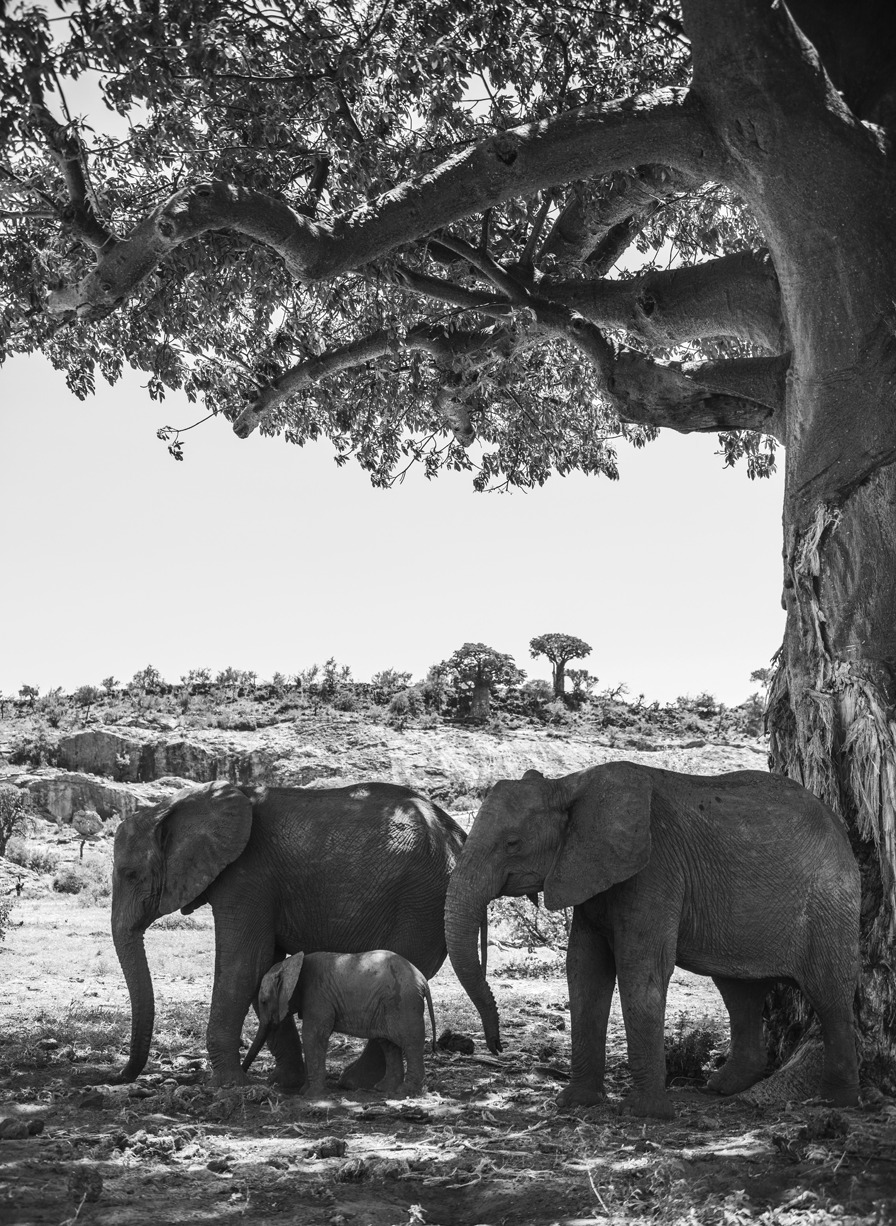
The Baobab tree. The relationship between a group of African elephants and a Baobab tree strains as droughts strike. Attribution: Samantha Kreling

Samantha Kreling from the University of Washington captured the winning image for this category. Here Samantha explains the photo shows “a group of African elephants shelter from the sun under a large Baobab tree as droughts strike Mapungubwe National Park in South Africa. On the tree, wear marks show where the elephants have stripped the bark to seek out water.” The Baobab tree inhabits the seasonal deserts of southern Africa where it can live for more than 2000 years. This ancient tree has adapted to its extreme environment by storing water in its barrel-like trunk when water availability is low. Sadly, recent research shows that these trees are victims of climate change. Elephants have long gouged water from the trunks of these fast-healing trees, but as temperatures rise, the elephants are now doing more damage than the trees can cope with. The black and white image highlights the need for action to prevent the permanent loss of these iconic trees (Fig. 4).

## Biodiversity under threat: runner-up

**Fig. 5:**
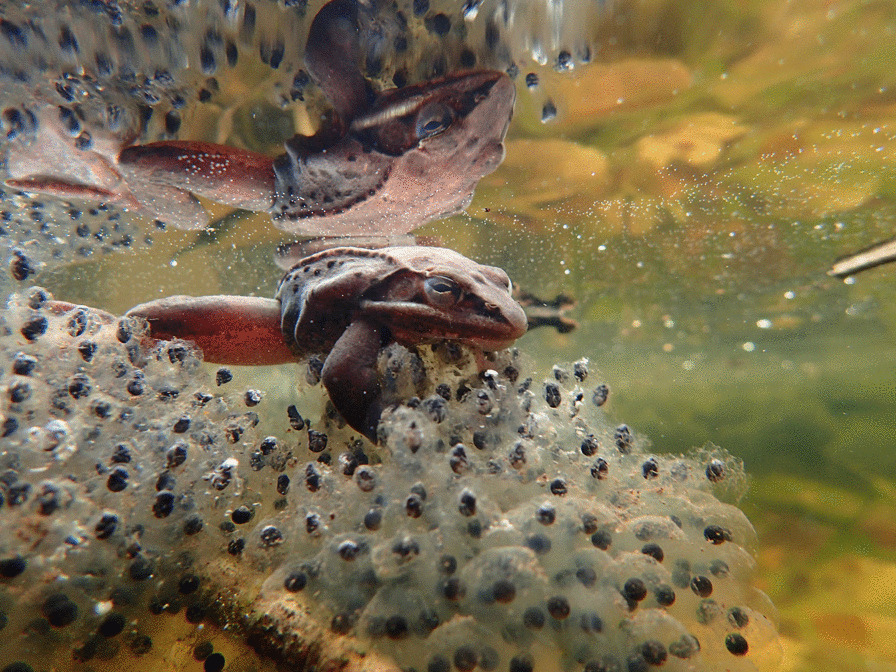
Wood frog under a freeze. A false spring—climate change threatens wood frog offspring. Attribution: Lindsey Swierk

Lindsey Swierk, an Assistant Research Professor at Binghamton University (State University of New York), submitted the runner-up for this category. Spring in the northeastern United States is arriving earlier and becoming more variable in temperature. This change poses a threat to many spring-breeding amphibians, including wood frogs. Lindsey explains, "Wood frogs (*Rana sylvatica*) are early spring breeders in temperate North America and congregate in vernal pools soon after the ice melts to mate and produce egg masses. Lately, wood frogs are breeding earlier in the year as climate change unseasonably warms early spring in the Northeastern USA. Unfortunately, winter storms can still catch frogs unexpectedly and trap them under the ice. Here, a male wood frog clings to an egg mass produced before a freeze; both the egg mass and the frog were recently trapped under ice. The frog survived, but many of the eggs did not." Senior Editorial Board Member Josef Settele comments, "I think it is important to realise that effects of major drivers of biodiversity change can also happen in counter-intuitive ways. In the present case, due to climate warming, there is an increased risk of the frog offspring dying because of cold/freezing (due to severe changes in phenology)" (Fig. 5).

## Life close up: best in category

**Fig. 6:**
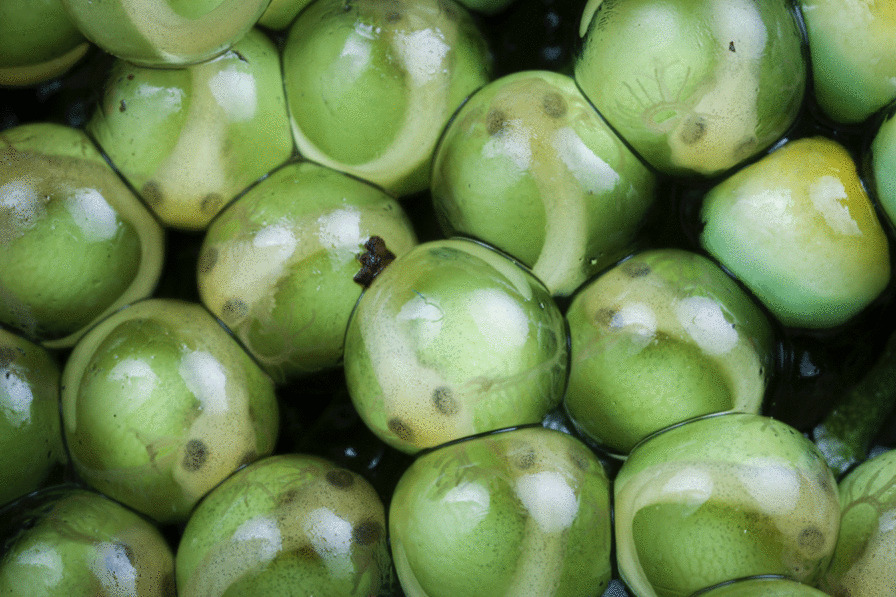
In ovo. Gliding treefrog siblings at an early stage of their development. Attribution: Brandon André Güell

The winner of the 'Life close up' category was taken by Brandon André Güell, a Costa Rican-American PhD student researching the developmental and behavioural ecology of gliding treefrogs at Boston University, USA. Brandon explains: "Pictured are gliding treefrog siblings, *Agalychnis spurrelli*, at an early stage of their development within their eggs. The embryos' bodies are clearly visible and distinct from their large green yolks and transparent external gills. This image also captures the details of individual melanophores and yolk veins, which are becoming apparent on the dorsal sides of embryos' bodies and yolks. These eggs were laid after a torrential wet-season rainstorm triggered an explosive breeding event on Costa Rica's Osa Peninsula. During these events, thousands of gliding treefrogs come together to reproduce and leave behind hundreds of thousands of eggs, most of which die from desiccation, predation, and fungal infection. Some eggs never develop, presumably because they are unfertilized and remain embryo-less and yellow in colour, as also pictured here. If undisturbed, after 6 days of development, the eggs will hatch. However, gliding treefrog eggs are particularly susceptible to predation because of their abundance and sessile nature and to desiccation because of their thin monolayered clutches that lack a gelatinous jelly core that aid in maintaining egg and clutch hydration in other closely related species. These eggs are not helpless, however. Hatching in gliding treefrogs is an excellent example of adaptive plasticity and environmentally cued hatching; embryos can hatch prematurely to escape predators, flooding, desiccation, and other egg threats" (Fig. 6).

## Life close up: runner-up

**Fig. 7:**
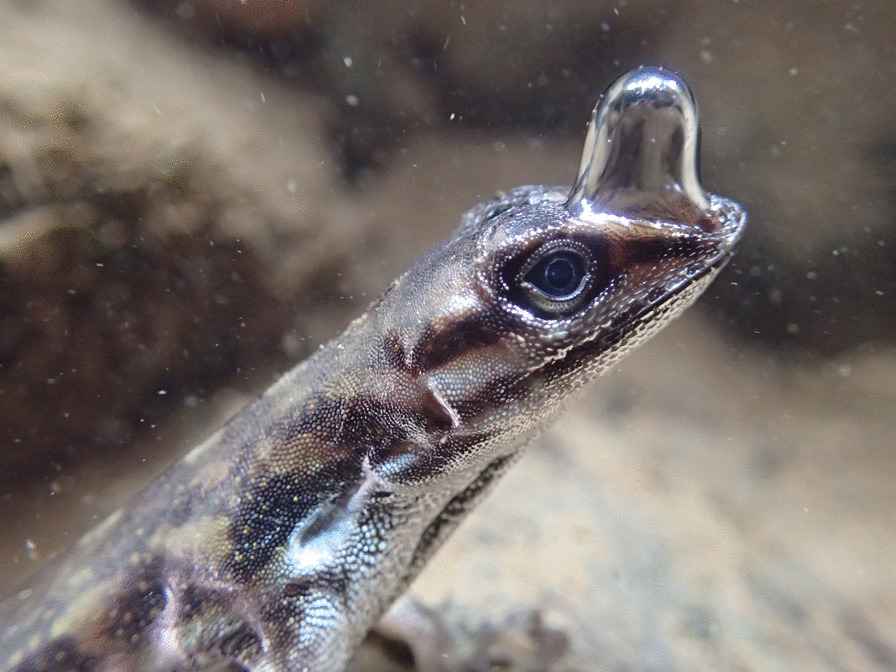
Bubble breathing in Water Anoles. An anole lizard dives using a clever trick to breathe underwater. Attribution: Lindsey Swierk

A photo of an anole lizard diving using a clever trick to breathe under water is the runner-up for this category, which was also captured by Lindsey Swierk. Lindsey comments that “Water Anoles (*Anolis aquaticus*) are small Neotropical lizards that escape to the water when threatened by predators. They can spend almost 20 min underwater, inhaling and exhaling a bubble of air that clings to their snout. Oxygen from this bubble is depleted over the underwater dive, which likely helps water anoles remain underwater for so long” (Fig. 7).

## Research in action: best in category

**Fig. 8:**
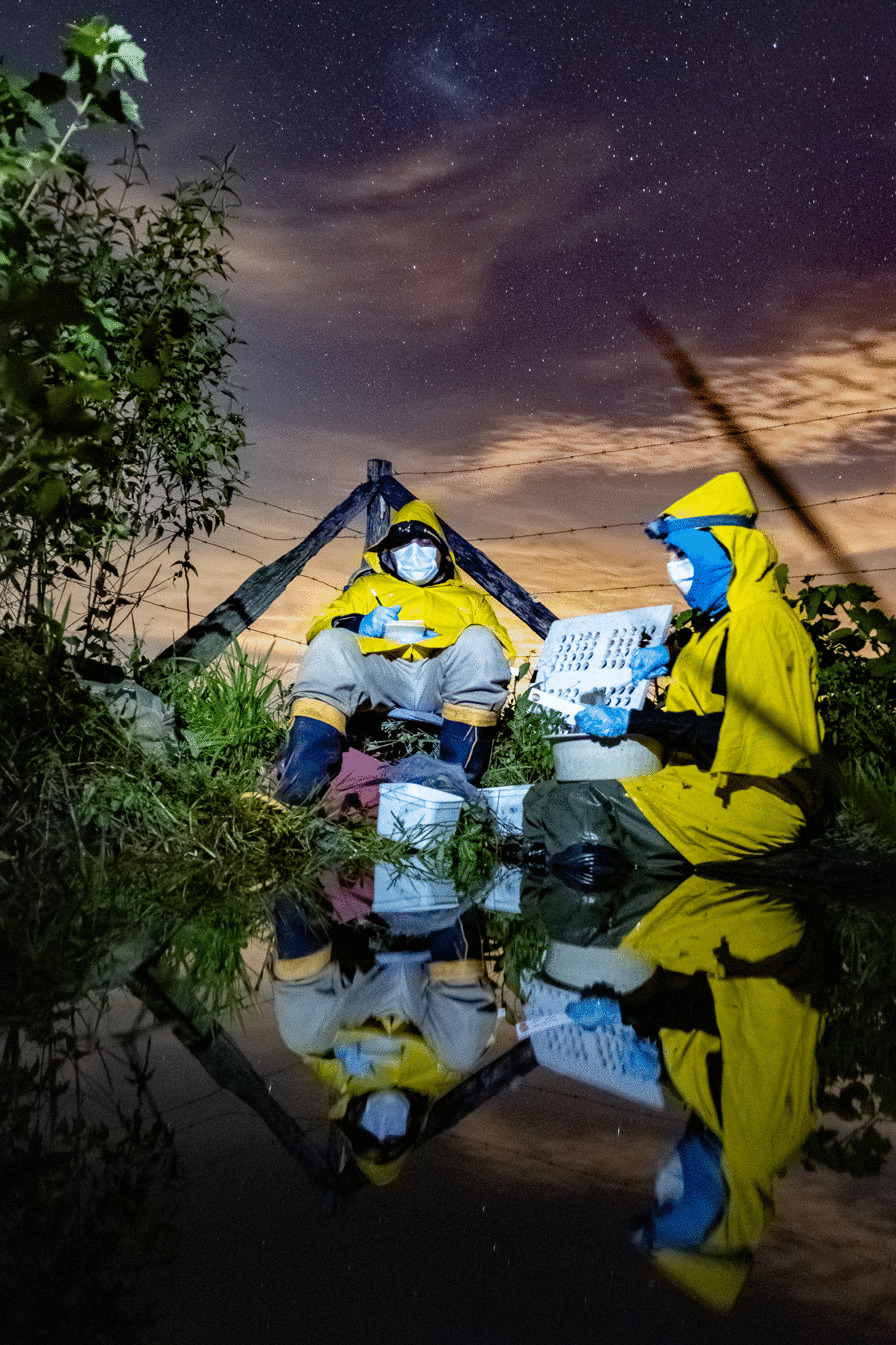
Fieldwork with masks, rain, and tadpoles. Researchers investigate the effect of isolated trees and land use on tadpole-mediated nutrient recycling during the COVD-19 pandemic. Attribution: Jeferson Ribeiro Amaral

The winning image for “Research in action” was submitted by Jeferson Ribeiro Amaral, a biologist working at Cornell University. Jeferson explains that the photo “represents the strength of two Ph.D. researchers from the State University of Rio de Janeiro performing fieldwork in the middle of the COVID-19 pandemic during thunderstorms.” Jeferson took this photo “while working as a research technician in the Laboratory of Rivers and Streams Ecology at the State University of Rio de Janeiro, Brazil”. He explains that “the PhD students present in the frame were investigating whether the presence of scattered trees could buffer the anthropogenic effects created by agricultural land use by increasing the abundance of frogs and positively affecting nutrient recycling inside ponds.” The photo clearly demonstrates the dedication and tireless resolve necessary in order to better our understanding (Fig. 8).

## Research in action: runner-up

**Fig. 9:**
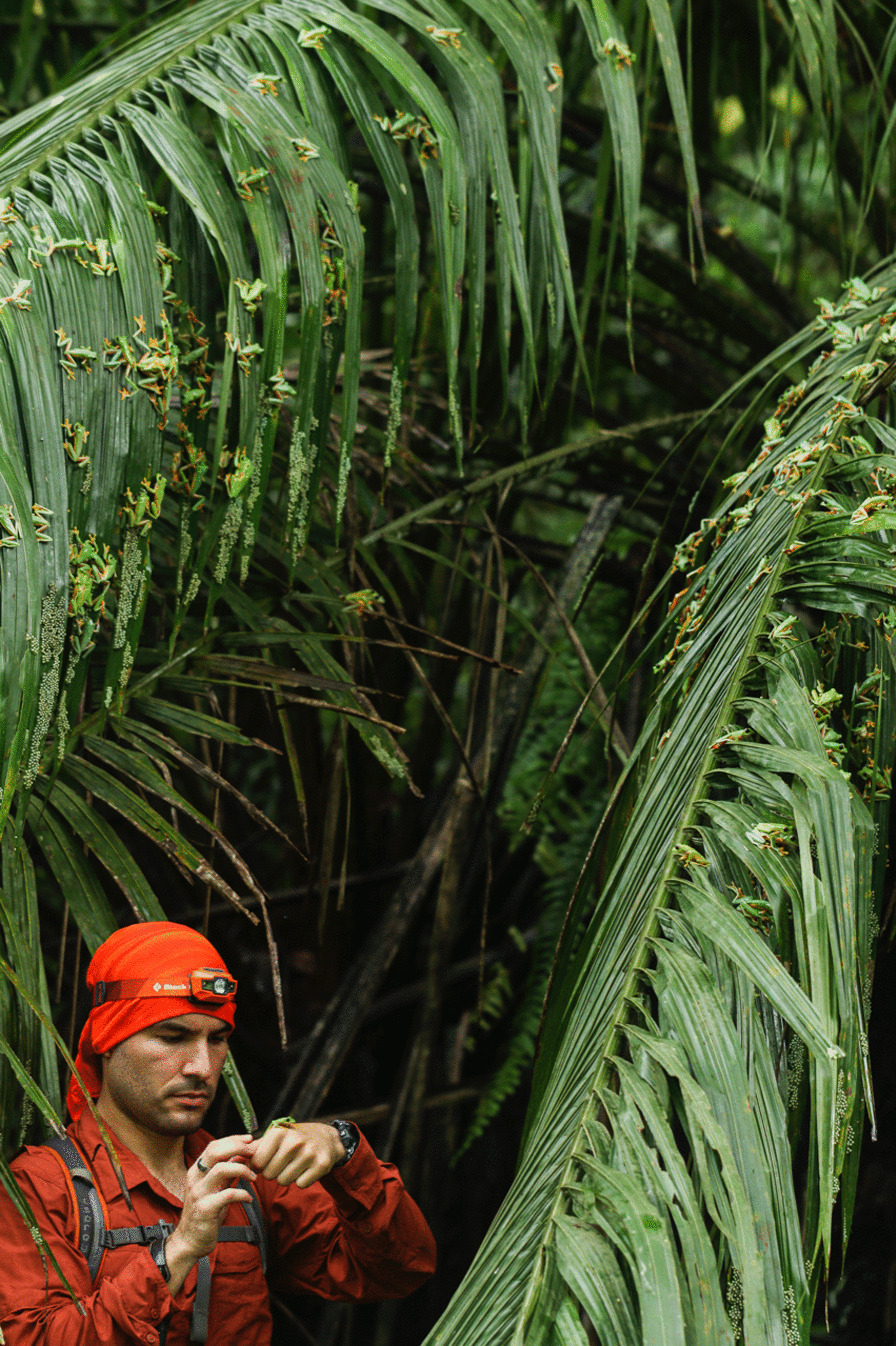
Focus amidst the chaos. PhD student, Brandon A. Güell, amidst thousands of reproducing gliding treefrogs

An image submitted by Brandon A. Güell was also selected as the runner-up for the 'Research in Action' category. The photo captures Brandon amidst thousands of gliding treefrogs, *Agalychnis spurrelli*, and their recently laid eggs on palm fronds. Brandon said: "This photo captures a treasured memory of the first explosive breeding event I observed, photographed, and collected data from for my dissertation research. Gliding treefrogs are of particular scientific and personal interest because of their understudied arboreal explosive breeding strategy and the diverse behaviours that may affect adult reproductive success. Moreover, hatching in gliding treefrogs is an excellent example of adaptive plasticity and environmentally cued hatching; embryos can hatch prematurely to escape predators, flooding, desiccation, and other egg threats. My dissertation research over the last five years aims to better understand this species' reproductive, behavioural, and developmental ecology using observation and experimentation in the field where they live. In this picture, I am standing waist-deep in a lowland tropical rainforest pond on Costa Rica's Osa Peninsula, holding an adult male gliding treefrog from which I'll later take measurements. That same day, and so many days after that, across four breeding seasons, I collected data on breeding phenology and its environmental triggers, adult reproductive behaviours, predator–prey interactions, embryo development and behaviours, among other things” (Fig. 9).

## Highly commended

Beyond our winning images we received many other fantastic photos. Our favourites are showcased in Additional file 1, 2 and 3.

## Conclusions

Thank you to everyone who helped make this year's annual image competition a success. We received many outstanding photos making judging a challenging but wonderful experience for all editors involved. We hope that our readers enjoy viewing these images and discovering the stories behind them. We look forward to you joining us for next year's photographic celebration of ecology and evolutionary biology.

## Supplementary Information

**Additional file 1**: **Fig S1. **Fluorescent Fungi. Bioluminescent fungi observed in the Bornean rainforest. Attribution: Julian Schrader.

**Additional file 2**: **Fig S2. **Deadly sight. A seabird’s stomach full of plastic waste. Attribution: Marine Cusa.

**Additional file 3**: **Fig S3**. Egg candling - looking for a glow of life. Researchers monitor the Bermuda petrel to help protect this endangered seabird. Attribution: Letizia Campioni.
