# Supplementary material for: 2022 BMC Ecology and Evolution image competition: the winning images
Source: BMC Ecol Evol. 2022 Aug 19;22:99. doi: 10.1186/s12862-022-02049-y (PMC9388214; doi:10.1186/s12862-022-02049-y)
Supplement: Supplementary file 1 — Additional file 1: Fig S1. Fluorescent Fungi. Bioluminescent fungi observed in the Bornean rainforest. Attribution: Julian Schrader. [file 12862_2022_2049_MOESM1_ESM.docx]

**Highly commended**

Beyond our winning images we received many other fantastic photos. Our favourites are showcased below.


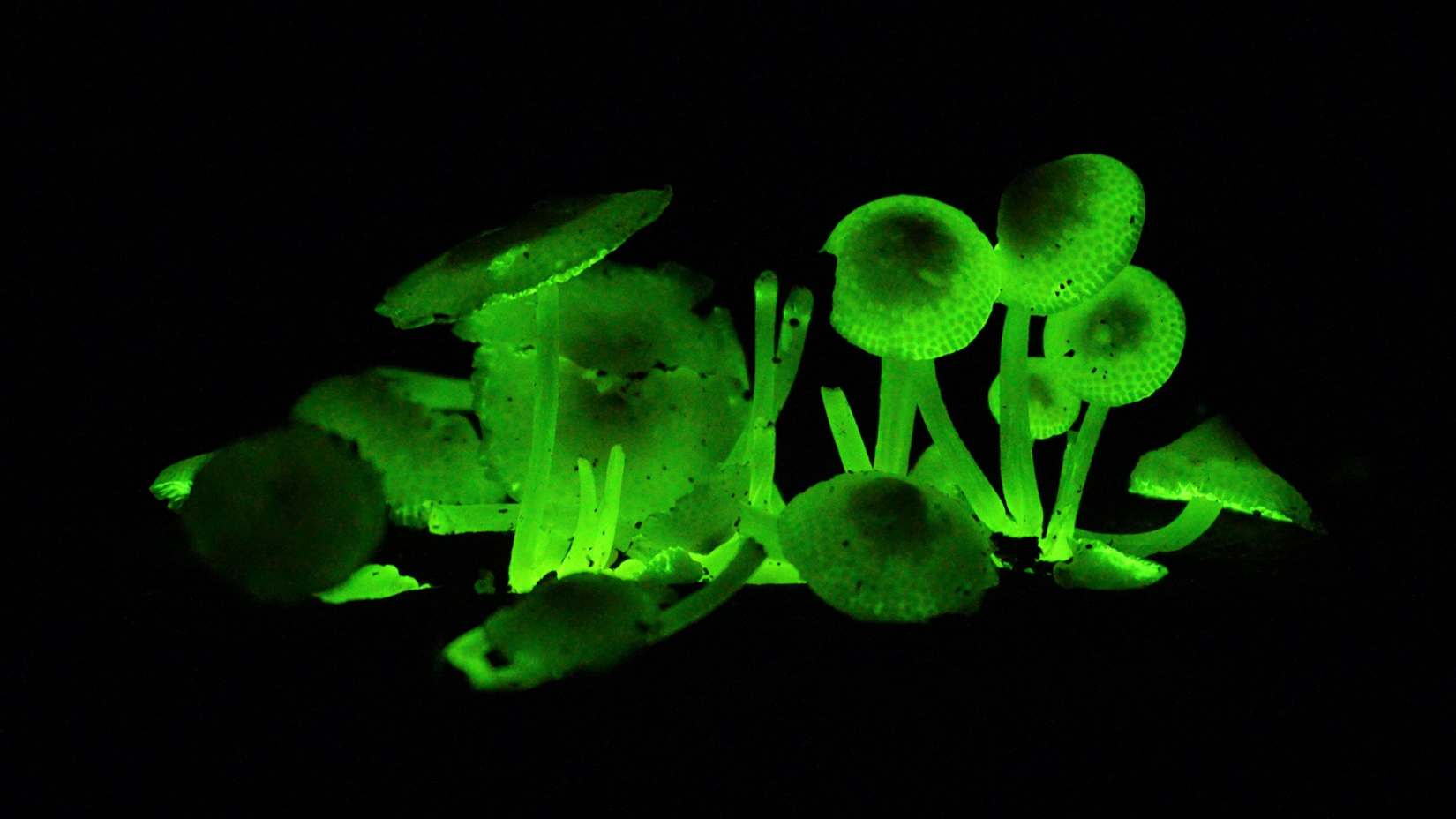


**Additional Fig 1: Fluorescent Fungi. Bioluminescent fungi observed in the Bornean rainforest. Attribution: Julian Schrader.**

Julian Schrader, an ecologist working at Macquarie University, Australia, submitted this highly commended image. Julian comments on the moment he captured this photo “The Bornean rainforests are full of wonders. Doing a hike after dusk revealed some new aspects to me I had never seen before. The forest floor was glowing with fluorescent mushrooms. This little assembly was especially beautiful and bright enough to be caught on camera. Why these fungi use bioluminescence is still not fully understood.*”* The threads of certain fungi form a glowing underground network, but the reason why they emit light in the darkness remains a mystery. Historically, fungi bioluminescence was written off as a bi-product of evolution with no function. However, the observation that some fungi only glow above ground led to the theory that it attracts insects which may help disperse fungal spores. This photo depicts one of nature’s remaining secrets, there is no one answer to why certain

fungi emit light, and it is fair to speculate that different species may glow for different reasons.
