# Supplementary material for: 2022 BMC Ecology and Evolution image competition: the winning images
Source: BMC Ecol Evol. 2022 Aug 19;22:99. doi: 10.1186/s12862-022-02049-y (PMC9388214; doi:10.1186/s12862-022-02049-y)
Supplement: Supplementary file 2 — Additional file 2: Fig S2. Deadly sight. A seabird’s stomach full of plastic waste. Attribution: Marine Cusa. [file 12862_2022_2049_MOESM2_ESM.docx]

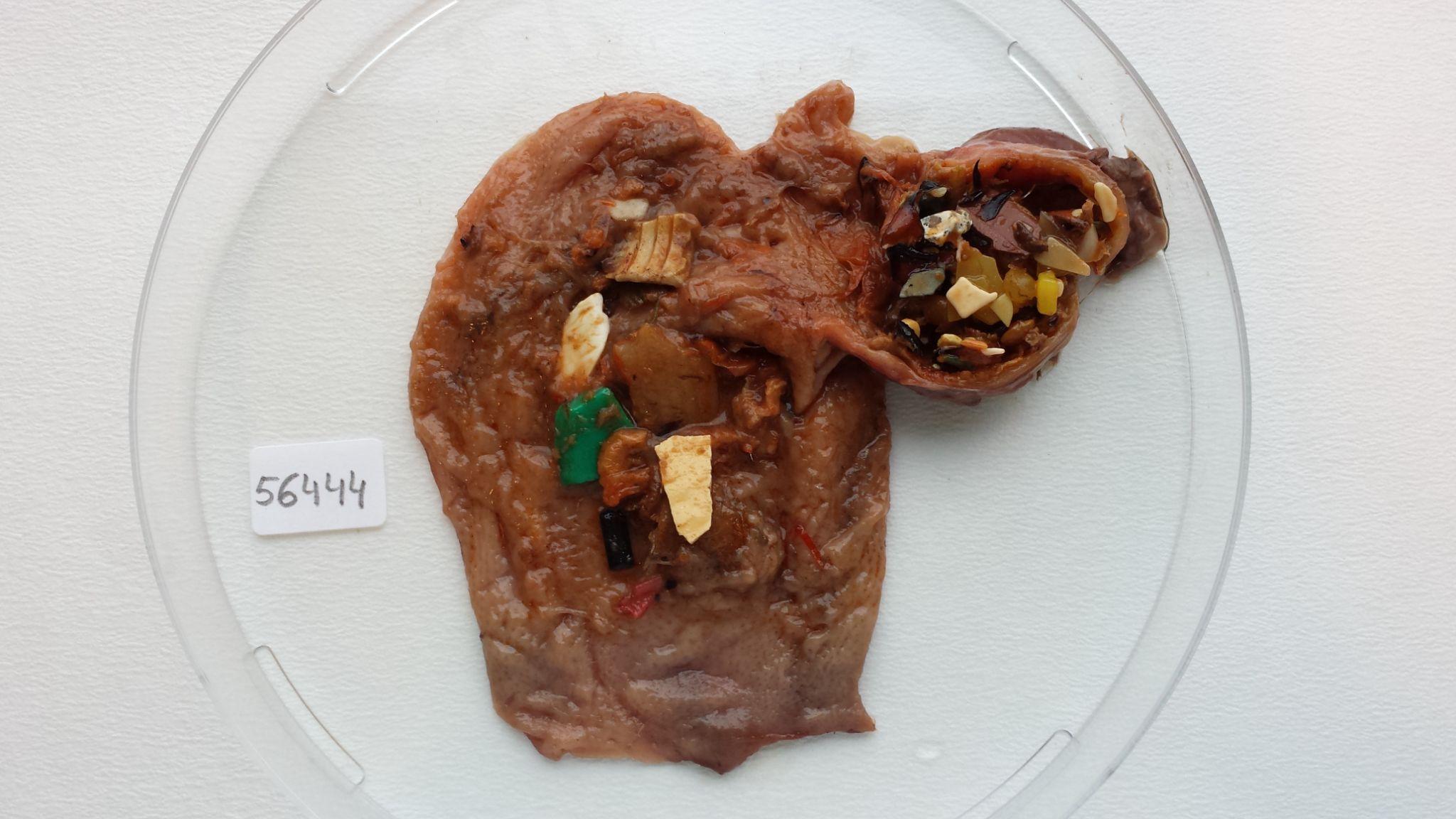


**Additional Fig 2: Deadly sight. A seabird’s stomach full of plastic waste. Attribution: Marine Cusa.**

This second highly commended image provides a stark reminder of the wide-reaching impact of human activity on the planet. The image was submitted by Marine Cusa, who recently obtained her PhD from the University of Salford, UK, where she explored how genetic tools can be used to prevent seafood fraud. Previously Marine worked as a Research Assistant for Aarhus University in Denmark where she was tasked to investigate the diet of seabirds from the coast of Greenland. It was during this time that Marine took this photo of the stomach of a northern fulmar (*Fulmarus glacialis*), a seabird from West Greenland, which was full of plastic fragments rather than prey. Marine said, “Plastic pollution is having a profound effect on ecosystems. Plastic clogs up the digestive system of the birds and are often transferred to juveniles through regurgitation, preventing them from acquiring the necessary calories for growth and survival.” Marine further comments that the stomach “in the photo was particularly distressing, with its gizzard looking like it was exploding with plastic. Of the 31 fulmars sampled, 28 had ingested plastic. This seabird diet investigation turned out to be quite a devastating one.” This image highlights the threat of plastic pollution to marine ecosystems.
