# Supplementary material for: 2022 BMC Ecology and Evolution image competition: the winning images
Source: BMC Ecol Evol. 2022 Aug 19;22:99. doi: 10.1186/s12862-022-02049-y (PMC9388214; doi:10.1186/s12862-022-02049-y)
Supplement: Supplementary file 3 — Additional file 3: Fig S3. Egg candling - looking for a glow of life. Researchers monitor the Bermuda petrel to help protect this endangered seabird. Attribution: Letizia Campioni. [file 12862_2022_2049_MOESM3_ESM.docx]

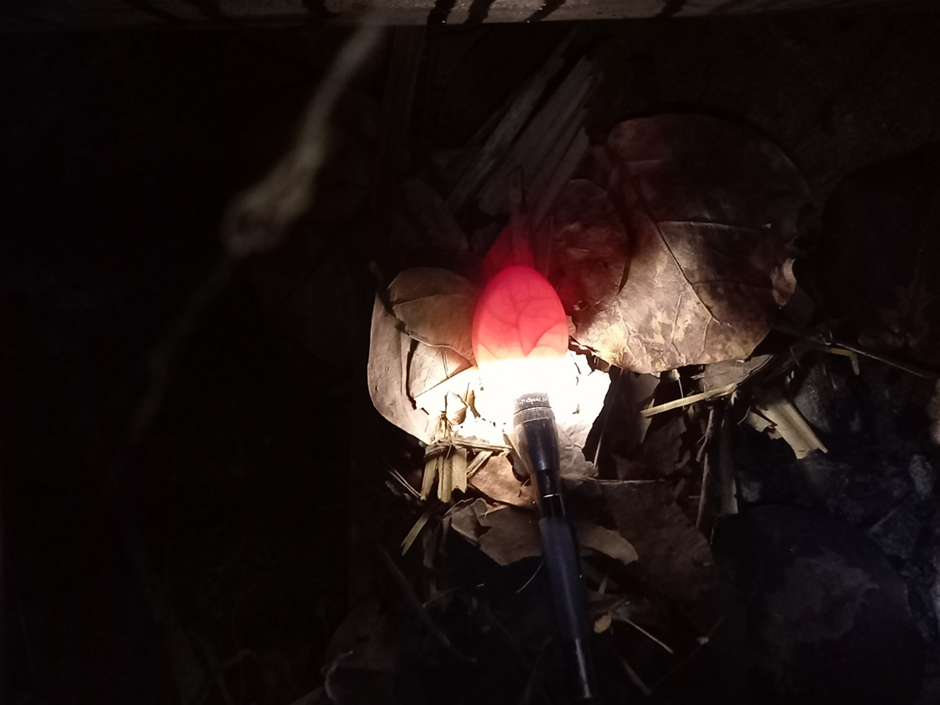


**Additional Fig 3**: **Egg candling - looking for a glow of life. Researchers monitor the Bermuda petrel to help protect this endangered seabird. Attribution: Letizia Campioni.**

The interesting story behind this photo submitted by Letizia Campioni, a postdoctoral researcher at the Marine and Environmental Sciences Centre (MARE) and ISPA - Instituto Universitário, Portugal also attracted a number of votes. Letizia explains, **“**Reproduction is a critical stage in animals’ annual cycle since it provides individuals with the opportunity to produce offspring some of which will make up the future breeding pool. For endangered species with small populations, such a life-history event is even more important since the recovery of the population will be highly dependent on the current investment that individuals will make in reproduction. This is particularly relevant in long-lived species with delayed breeding where immature individuals can recruit when 4-9 years old. The Bermuda petrel, *Pterodroma* *cahow,* is a long-lived seabird species endemic to the Bermuda island where it nests in artificial burrows. In 1951 the Bermuda petrel was rediscovered after being considered extinct for more than 300 years. Currently the population counts only 155 breeding pairs, and the species is classified as endangered. Thanks to the implementation of a long-term conservation and recovery programme, over the last 60 years the original breeding population (ca. 18 breeding pairs) increased 10 times. As part of the monitoring scheme, every year, each nest and the single egg therein is monitored to check fertility and embryo development. Candling the egg in its early stages can be critical to inform conservation actions and research on this endangered species. The presence of well-developed vessels and the visible movements of the embryo are good signs. Knowing the fate of the egg at different stages of embryo development helped us to run a toxicological and comparative study analysing trace elements in eggshells.”
